# Management of Complex Avulsion Injuries of the Dorsum of the Foot and Ankle in Pediatric Patients by Using Local Delayed Flaps and Skin Grafts

**Published:** 2010-10-13

**Authors:** Ahmed Elshahat

**Affiliations:** Plastic Surgery Department, Ain Shams University, Cairo, Egypt

## Abstract

**Objective:** Avulsion injuries of the dorsum of the foot and ankle present difficult reconstructive problems especially in pediatric patients. Usually, there is exposure of bones, joints, tendons, or ligaments, which requires coverage by flaps. The best skin for coverage is the local skin, but the remaining intact skin is usually limited. Usage of such local skin necessitates elevation of long and narrow skin flaps. These flaps need delay to survive. **Method:** This study included 8 children who sustained avulsion injuries to their feet and ankles in road traffic accidents. Debridement and delaying flaps using bipedicled flap elevation technique were done in the first session. Dressing of the raw areas was done while the flaps were being delayed. The delayed flaps were elevated in the second session to cover any exposed bone, joints, or ligaments. Split thickness skin grafts were applied to the donor site and to the granulating raw areas. **Results:** Complete survival of the flaps and complete take of the skin grafts with minimal donor site morbidity. **Conclusion:** This technique of delaying flaps from the intact skin adjacent to the defect is safe, successful and allows minimal hospital stay.

The skin of the dorsum of the foot and ankle is vulnerable to avulsion trauma, because it is thin and loosely attached to the underlying tendons, ligaments, and bones.^1^ Injury usually involves exposure of tendons, ligaments, joints or bones which compromises the take of skin grafts if used alone for coverage. Flaps used for reconstruction includes local flaps, regional flaps, or free flaps. The best are the local flaps, because Gillies often wrote that “the next best skin is the next nearest skin.”^2^ One of the obstacles to the use of the local flaps is the limited remaining intact skin on the dorsum of the foot. The remaining intact skin is usually narrow and any flap needs to be long enough to reach the exposed joints, bones, or tendons. The best way to elevate such flaps without compromising the vascularity is to delay these flaps.^3^,^4^ The aim of this work is to simplify the management of complex avulsion injuries of foots and ankles in pediatric patients by using delayed flaps from the remaining intact skin at the dorsum of the foot.

## PATIENTS AND METHODS

**Figure 1:**
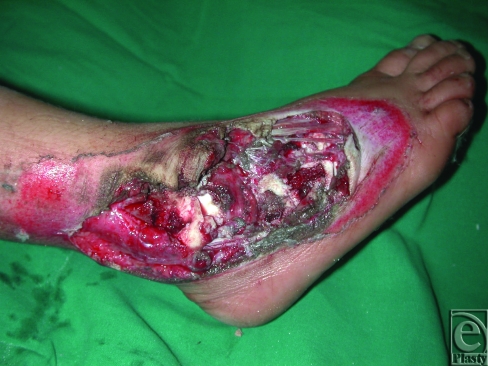
Complex avulsion injury of the dorsum of the right foot and ankle in an 8-year-old child.

**Figure 2:**
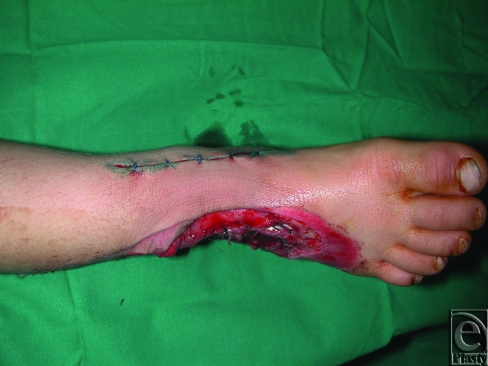
The design of delayed flap in the same patient in Figure 1.

Eight pediatric patients who sustained avulsion injuries to their foot and ankle in road traffic accidents were managed. They were 7 male and 1 female. They ranged in age between 4 and 12 years. They presented with avulsion injuries involving the dorsum of the foot and lateral ankle regions. There was fracture of lower tibia and fibula in one case that necessitated external fixation. There were different combinations of exposure of bones, tendons, ligaments, and joints. The edges of the wounds showed friction burns. The preoperative photo of one of the patients on admission is shown in Figure 1. After adequate debridement, design of delay of 1 or more local flaps needed for coverage of exposed bones, tendons, ligaments, or joints was performed. Delay of only 1 flap was performed in 5 cases and delay of 2 flaps in the same patient was done in the remaining 3 patients. The delay was in the form of bipedicled flap by incising the flap parallel to the edge of the defect. The flap was undermined to cut the underneath perforators. The length of the flap was double the width of any single pedicle or equal to the sum of both pedicles. The bipedicled flaps were resutured again in their place (Figs 2, 4, and 5) and the wounds were covered with Vaseline gauze and dressings. The ankles were positioned in 90 degree, using splints except in the patient who had external fixation. The patients were discharged the day after the day of surgery and follow-up dressings were performed in outpatient clinics every 2 days. After 2 weeks, the patients were readmitted to the hospital for definite coverage. Under general anesthesia, the delayed flaps were elevated on either the proximal or distal pedicle according to the need. This provided flaps with length to width ratio 2 to 1. The flaps were rotated to cover the exposed bones, tendons, ligaments, or joints. The donor sites of the flaps and the granulating raw areas were covered with split-thickness skin grafts harvested from the contralateral thighs and tie-over dressings were applied over the skin grafts. The patients were discharged the next day for follow-up in outpatient clinics. The first dressing was done on the third postoperative day. The posterior splints remained for at least 2 weeks. The follow-up period ranged from 6 months to 1 year.

## RESULTS

**Figure 3:**
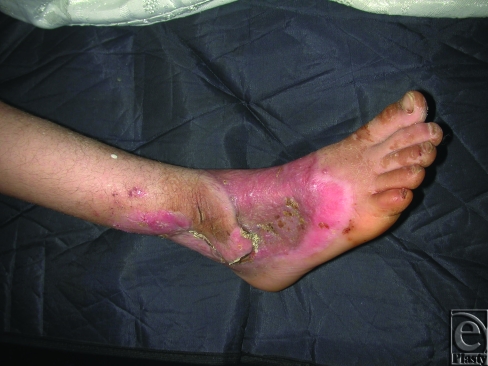
A postoperative photo of the same child in Figure 1, 1 month after coverage using delayed flap from the remaining intact skin on the medial aspect of the dorsum of foot and ankle. The donor site of the flap and the remaining raw area were covered with split-thickness skin grafts.

**Figure 4:**
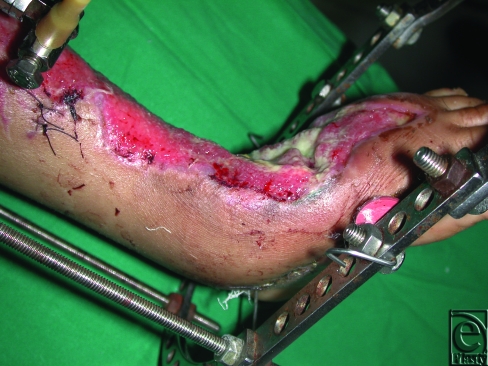
Complex avulsion injury of the dorsum of the foot and ankle and lower leg in a 5-year-old child. External fixation is shown. The remaining intact skin on the medial aspect of the dorsum of the foot and the most posterior extension of the incision used for delay are shown.

**Figure 5:**
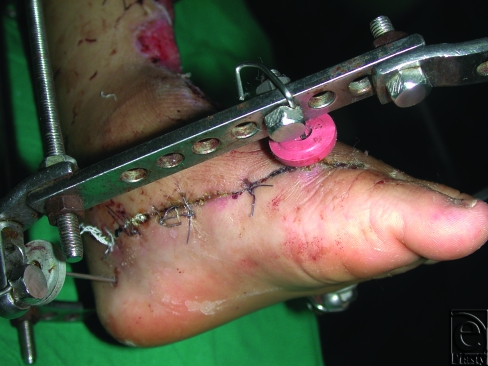
The design of delayed flap in the same patient in Figure 4.

**Figure 6:**
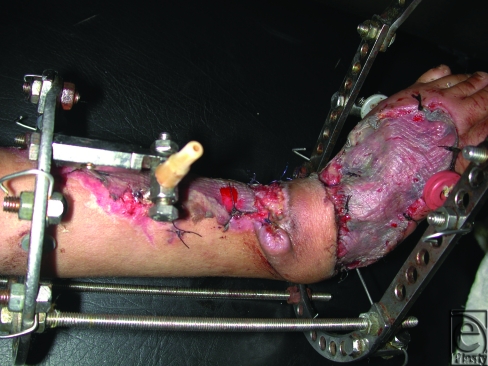
A postoperative photo of the same child in Figure 4 after 5 days of insetting the delayed flap to cover the exposed ankle joint. Split-thickness skin grafts were applied to the donor site of the flap and to the raw areas.

The 2-week delay in coverage of the raw area allowed granulation of most of the bed except exposed bone at the ankle joints. The 2-week gap in addition allowed healing of the friction burns at the edges of the wound. The delayed flaps survived completely and provided adequate coverage. The take of the split-thickness skin grafts over the donor site of flaps and over the granulated raw area was 100%. The donor site of split-thickness skin grafts healed in 2 weeks. The splints provided stability of the ankle joint in a right-angle position and prevented shearing of the split-thickness skin grafts. Loss of the dorsi-flexors of the lateral toes in trauma did not constitute a major functional deficit. The aesthetic outcome was acceptable because there was no donor site morbidity in any region away from the site of injury. Apart from the patient who had external fixation, the patients became ambulatory after complete wound healing. No subsequent breakdown of the grafted skin or unstable scars with regular wearing of shoes were found. There were hypertrophic scars in 3 patients, which necessitated wearing pressure garments for 3 months. The patients walked without noticeable limping after 2 to 3 months. The relatively short follow-up period did not allow investigating the effect on growth (limb-length discrepancy). The postoperative photos of 2 children are shown in Figures 3 and 6.

## DISCUSSION

Reconstruction of the soft tissue defect on the dorsum of the foot and ankle is challenging. Local skin flaps are limited by the area of the remaining nonavulsed skin on the dorsum of the foot. Reverse-flow fasciocutaneous flaps have the disadvantages of sacrificing an important artery in the leg and the obvious contour deformity of the donor site.^5^ Distally based adipofascial flaps are thin and do not necessitate sacrifice important artery and leave no donor site contour deformity, but venous congestion may occur.^6^ Cross-leg flaps are out of favor because of difficulties in immobilizing both extremities in bulky casts or bandages. Mooney et al^7^ in 1998 introduced the use of external fixation to stabilize cross-extremity flaps in pediatric patients. But cross-leg flaps result in aesthetically poor donor site. Free flaps are bulky, require expertise, and are time consuming.^1^ They create a remote donor area and disturb major vessels of an already traumatized limb.^8^,^9^ Vacuum-assisted closure therapy has been shown to assist healing by secondary intention,^10^ but healing by secondary intention needs long time.

The remaining intact skin on the dorsum of the foot can be used for reconstruction, but the risk of impaired vascularity is high because the flap will be long and narrow. To avoid this risk, the flap was delayed. A surgical delay increases the survival length of a flap by allowing the choke vessels to dilate between adjacent perforators.^3^,^4^

The advantages of the local delayed flaps are numerous. They include safe flap elevation regarding vascularity, no donor site morbidity in leg, and no flap crossing the ankle joint. One sheet graft can be applied to both raw area and the adjacent donor site of flap. It is a narrow and thin skin flap and will not add any bulk or produce obvious dog ears. The contracture, which is expected to happen in skin graft on the long term, compensates for the loss of dorsiflexors of lateral toes.

This technique is advised to be used by surgeons who do not have free-flap expertise or who cannot achieve high success rate. Thin free cutaneous flaps surely will provide total coverage with no need for skin graft and better local aesthetic results.
